# Potential therapeutic mechanism of deep brain stimulation of the nucleus accumbens in obsessive-compulsive disorder

**DOI:** 10.3389/fncel.2022.1057887

**Published:** 2023-01-04

**Authors:** Yifeng Shi, Mengqi Wang, Linglong Xiao, Luolan Gui, Wen Zheng, Lin Bai, Bo Su, Bin Li, Yangyang Xu, Wei Pan, Jie Zhang, Wei Wang

**Affiliations:** ^1^Department of Neurosurgery, West China Hospital, Sichuan University, Chengdu, Sichuan, China; ^2^Laboratory of Clinical Proteomics and Metabolomics, Frontiers Science Center for Disease-Related Molecular Network, National Clinical Research Center for Geriatrics, West China Hospital, Institutes for Systems Genetics, Sichuan University, Chengdu, Sichuan, China; ^3^Histology and Imaging Platform, Core Facilities of West China Hospital, Sichuan University, Chengdu, Sichuan, China; ^4^Key Laboratory of Transplant Engineering and Immunology, West China Hospital, Sichuan University, Chengdu, Sichuan, China; ^5^Mental Health Center, West China Hospital, Sichuan University, Chengdu, Sichuan, China

**Keywords:** deep brain stimulation, therapy, nucleus accumbens, obsessive-compulsive disorder, electrophysiology *in vivo*, neurotransmitters

## Abstract

Deep brain stimulation (DBS) of the nucleus accumbens (NAc) (NAc-DBS) is an effective solution to refractory obsessive-compulsive disorder (OCD). However, evidence for the neurobiological mechanisms of OCD and the effect of NAc-DBS is still lacking. One hypothesis is that the electrophysiological activities in the NAc are modulated by DBS, and another hypothesis is that the activities of neurotransmitters in the NAc are influenced by DBS. To investigate these potential alterations, rats with quinpirole (QNP)- induced OCD were treated with DBS of the core part of NAc. Then, extracellular spikes (SPK) and local field potentials (LFP) in the NAc were recorded, and the levels of relevant neurotransmitters and related proteins were measured. Analysis of SPK revealed that the firing rate was decreased and the firing pattern was changed after NAc-DBS, and analysis of LFP showed that overall power spectral density (PSD) levels were reduced after NAc-DBS. Additionally, we found that the relative powers of the theta band, alpha band and beta band were increased in OCD status, while the relative powers of the delta band and gamma band were decreased. This pathological pattern of power distribution was reformed by NAc-DBS. Furthermore, we found that the local levels of monoamines [dopamine (DA) and serotonin (5-HT)] and amino acids [glutamate (Glu) and gamma-aminobutyric acid (GABA)] in the NAc were increased in OCD status, and that the expression of the two types of DA receptors in the NAc exhibited an opposite change. These abnormalities could be reversed by NAc-DBS. These findings provide a more comprehensive understanding about the function of the NAc in the pathophysiology of OCD and provide more detailed evidence for the potential effect of NAc-DBS.

## Introduction

Obsessive-compulsive disorder (OCD) is a neuropsychiatric disease that involves obsessions or compulsions that cause distress or impair functioning (Hirschtritt et al., 2017). The prevalence of OCD is 1–3% in general, and 30–40% of OCD patients develop into refractory OCD (Husted and Shapira, 2004; Groth, 2018). Refractory OCD seriously affects the lives of patients and their ability to work, and causes great pain and burden to patients and their families (Pinto et al., 2006; van Oudheusden et al., 2020). Therefore, identifying effective treatments for OCD is very important. As an alternative to medication, psychotherapy and physical therapy, deep brain stimulation (DBS) is gaining increasing attention because it is a reversible and titratable form of neuromodulation (Kumar et al., 2019; Goodman et al., 2020). The nucleus accumbens (NAc) is considered as the limbic-motor interface and plays an important role in brain networks related to motivation and reward processing (Groenewegen et al., 1996; Salamone et al., 2007; Nicola, 2010; Floresco, 2015). It has been suggested that dysfunction of the NAc is associated with various neuropsychiatric disorders (Mavridis, 2015; Park et al., 2019; Gendelis et al., 2021; Bayassi-Jakowicka et al., 2022). The NAc is one of the most commonly used neurosurgical targets in the treatment of OCD (Denys et al., 2010; Greenberg et al., 2010; Huys et al., 2019; Raviv et al., 2020), however, there are still many questions related to the use of DBS of the NAc (NAc-DBS) for the treatment of OCD, because the relevant underlying pathophysiological mechanisms are unclear, including the effect of NAc-DBS on neural electrophysiological activities and neurotransmitters activities.

**FIGURE 1:**
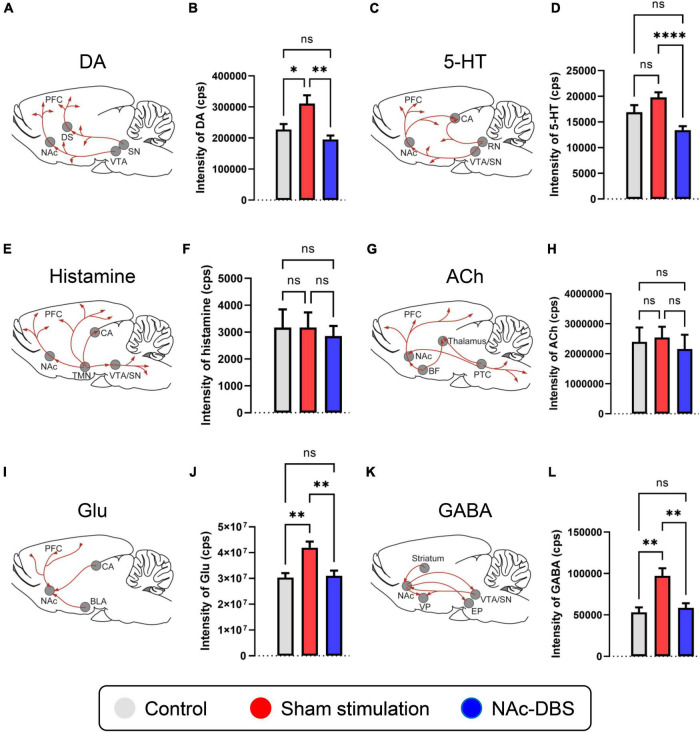
Changes in the levels of neurotransmitters of interest in the NAc in the control, sham stimulation and NAc-DBS groups. **(A)** Illustration of dopaminergic projections to the NAc. **(B)** The bar graph represents the mean level of the response intensity indicating the concentration of DA in the NAc. **(C)** Illustration of serotonergic projections to the NAc. **(D)** The bar graph represents the mean level of the intensity of 5-HT in the NAc. **(E)** Illustration of histaminergic projections to the NAc. **(F)** The bar graph represents the mean level of the intensity of histamine in the NAc. **(G)** Illustration of cholinergic projections to the NAc. **(H)** The bar graph represents the mean level of the intensity of ACh in the NAc. **(I)** Illustration of glutamatergic projections to the NAc. **(J)** The bar graph represents the mean level of the intensity of Glu in the NAc. **(K)** Illustration of the GABAergic pathway in the NAc. **(L)** The bar graph represents the mean level of the intensity of GABA in the NAc. All illustrations are modified from the stereotaxic atlas of Paxinos and Watson (6th edition). DA, dopamine; VTA, ventral tegmental area; 5-HT, serotonin; RN, raphe nuclei; TMN, tuberomammillary nucleus; ACh, acetylcholine; BF, basal forebrain; PTC, pontomesencephalo-tegmental complex; Glu, glutamate; BLA, basolateral amygdala; CA, hippocampus; GABA, gamma-aminobutyric acid; VP, ventral pallidum; SN, substantia nigra; EP, entopeduncular nucleus. The data are presented as the mean ± SEM; *n* = 11 vs. *n* = 23 vs. *n* = 21; ****P < 0.0001, **P < 0.01, *P < 0.05; ns, no significance.

Spikes (SPK) and local field potentials (LFP) measurements can be taken to assess neuronal firing characteristics and synchronous activity of membrane potentials in a large population of neurons (Buzsáki et al., 2012; Slutzky and Flint, 2017; Hagen et al., 2018; Gonzalez-Escamilla et al., 2020). There are only a few studies which reported some limited evidence on the LFP in OCD (McCracken and Grace, 2007; Neumann et al., 2014; Schüller et al., 2015; Miller et al., 2019), however, the LFP activities were mainly focused on cortex but not on deep NAc region, and the details of LFP and SPK in the NAc in OCD status and even in the DBS condition are still lacking. Moreover, changes in neurotransmitter activity may underlie OCD and the effect of DBS. In case of the NAc, its output patterns are relatively simple and mainly consist of gamma-aminobutyric acid (GABA)ergic projections to the substantia nigra (SN), ventral pallidum (VP), ventral tegmental area (VTA) and entopeduncular nucleus (EP) (Figure 1K) (Heimer et al., 1991; Usuda et al., 1998; Tripathi et al., 2010). However, as opposed to its simple output pattern which is mainly consisted of GABAergic projections, its input patterns are quite complicated, the NAc receives various input projections of many different neurotransmitters from wide brain regions, including dopaminergic, serotonergic, histaminergic, cholinergic, glutamatergic projections and so on. To be specific, the dopaminergic projections to the NAc mainly originate from the VTA (Figure 1A) (Hadley et al., 2014; Lammel et al., 2014; Cooper et al., 2017), the serotonergic projections to the NAc mainly originate from the raphe nuclei (RN) (Figure 1C) (Modell et al., 1989; Kiyasova et al., 2011; Alonso et al., 2013; Kim et al., 2019), histaminergic projections to the NAc mainly originate from the tuberomammillary nucleus (TMN) (Figure 1E) (Zhang et al., 2020; Manz et al., 2021), cholinergic projections to the NAc mainly originate from the basal forebrain (BF) and pontomesencephalo-tegmental complex (PTC) (Figure 1G) (Mark et al., 2011; Laurent et al., 2014; Luchicchi et al., 2014; Gielow and Zaborszky, 2017), and glutamatergic projections to the NAc mainly originate from the broad cortex, hippocampus (CA) and basolateral amygdala (BLA) (Figure 1I) (Walaas, 1981; Tarazi et al., 1998; Saul’skaya and Mikhailova, 2003; Zhu et al., 2022). Recently, some studies have revealed changes in the levels of these neurotransmitters and relevant receptors in OCD, and it is believed that the disturbances of these substances especially in the regions related to the cortico-striato-thalamico-cortical (CSTC) circuitry are normally considered as the basis of OCD (Winslow and Insel, 1990; Fineberg et al., 2011; van Dijk et al., 2012; Haleem et al., 2014; Pittenger, 2015; Ade et al., 2016; Vlček et al., 2018; Zhan et al., 2020; Zai, 2021). Nevertheless, research on the neurotransmitters that play a role in the NAc is lacking, and it is still not clear how the activity of these substances changes in OCD patients after NAc-DBS.

In the present study, a quinpirole (QNP)-induced OCD rat model was adopted, and the open field test (OFT) relevant to compulsive checking behavior and the elevated plus maze (EPM) were used for the behavioral assessment (Szechtman et al., 1998, 2017; Hoffman, 2011). Given that the core of the NAc mediates the control of goal-oriented behaviors and obsessive-compulsive-like behaviors (Di Chiara, 2002; Zhang et al., 2020), micro-electrodes were implanted into the NAc core for stimulation and recording. Then SPK signals were recorded to obtain information about neuronal firing patterns, including the firing rate and indicators of interspike interval (ISI), including the coefficient of variance (CV) and asymmetry index (AI) (Alam et al., 2012, 2015). On the other hand, LFP signals were recorded to obtain information about synchronous activity through analysis of time-frequency spectrograms and the power spectral density (PSD) (Geng et al., 2016; Salimpour et al., 2022; Wang et al., 2022; Yu et al., 2022). To analyze neurotransmitter activity, dopamine (DA), serotonin (5-HT), histamine, acetylcholine (ACh), glutamate (Glu) and GABA levels in location of the NAc were assessed through high-performance liquid chromatography (HPLC) combined with mass spectrometry (MS). Additionally, immunofluorescence staining for tyrosine hydroxylase (TH) and tryptophan hydroxylase-2 (TPH2) was performed to determine the activity of dopaminergic neurons in the VTA and serotonergic neurons in the RN, and DA receptor (DRD1 and DRD2) levels in the NAc were evaluated by immunohistochemical staining. In general, through the study from the aspects of neural electrophysiology and neurotransmitters, we aimed to provide more abundant evidence for the pathophysiological mechanisms of OCD and therapeutic mechanisms of NAc-DBS.

## Materials and methods

### Animals

A total of 120 male adult Sprague-Dawley rats (weighing 290–330 g, Huafukang Animal Centre, China) were used in this study. The rats were housed in transparent cages in a temperature- and humidity-controlled environment (25°C, 45%), on a 12 h light/12 h dark cycle, and *ad libitum* access to food and water was provided. All the animal experimental and care procedures were approved by the Biomedical Research Ethics Committee of West China Hospital (Protocol Number: 20211434A).

### Experimental design

**FIGURE 2:**
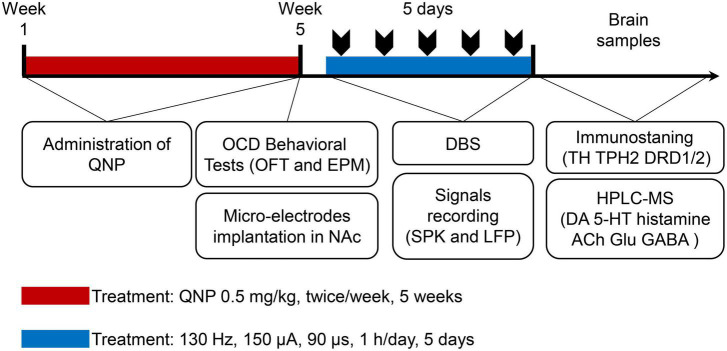
Graphical presentation of the timeline of the whole experiment design.

First, the rats were randomly divided into 2 groups: the control group (subcutaneous injection of saline) and the OCD group (administration of QNP). After OCD animal modeling and behavioral evaluation, the rats in the OCD group were randomly divided into 2 groups: the sham stimulation group (recording but no stimulation) and the NAc-DBS group (DBS at set parameters and recording). The whole procedure is shown in Figure 2.

### OCD animal model and behavioral assessment

The rats in the OCD group were given subcutaneous injections of QNP (Sigma-Aldrich, US, 0.5 mg/kg) twice a week for 5 weeks. The rats in the control group received subcutaneous injections of saline twice a week for the same time. During the last week, 10 min after each injection, the rats in both groups were subjected to the OFT relevant to compulsive checking behavior. The Visu Track system (XinRuan Technology, China) was used to monitor the rats for 30 min on a 100 × 100 cm table containing four 8 × 8 cm boxes (2 in the center area and 2 in the corner) as potential home bases. The home base was identified as the box with the highest number of visits, and the number of home base visits and total moving distance in the OFT were measured. In addition, the EPM was conducted to evaluated the condition of anxiety, the EPM was composed of two closed arms (50 × 10 cm) and two open arms (50 × 10 cm), the walls surrounded the closed arms were 30 cm high, and the maze was elevated 50 cm above the floor. The rats in each group were placed individually in the central zone of the EPM directing to one open arm, and then the Visu Track system was used to monitor the rats for 5 min. After the monitoring, the percentage of visit times to closed arms and the percentage of total spent time in the closed arms were calculated subsequently. The average measures from 2 experiments in the last week were used as the final results of behavioral evaluation.

### Electrode implantation

**FIGURE 3:**
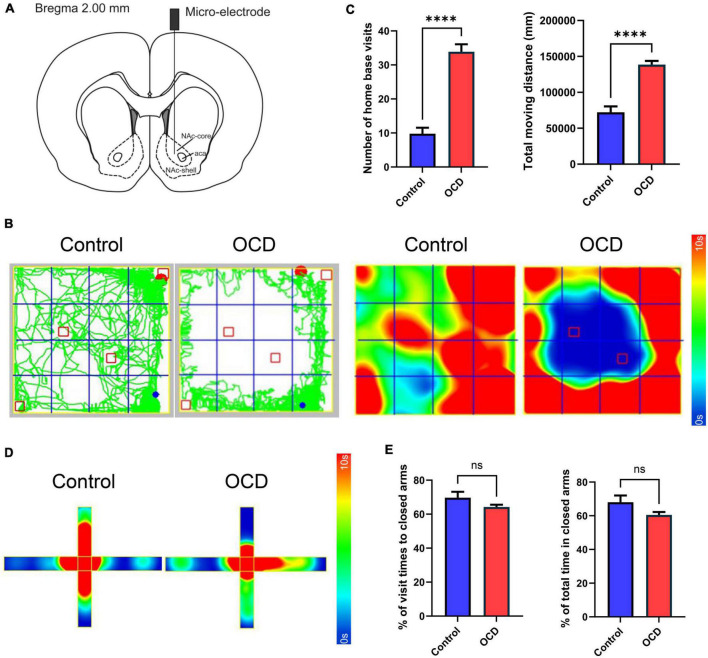
Location instruction and behavioral assessments. **(A)** Schematic diagram indicating the location of the micro-electrode in the NAc, modified from the stereotaxic atlas of Paxinos and Watson (6th edition). **(B)** Traces and 2D heatmaps of rats injected with QNP (OCD) and saline (control) in the OFT. **(C)** The bar graphs represent the level of number of home base visits and total moving distance in the OFT, by the control and OCD groups. **(D)** 2D heatmaps of rats in the EPM. The horizontal direction represents the closed arms, and the vertical direction represents the open arms. **(E)** The bar graphs represent the level of percentage of visit times to closed arms and percentage of total spent time in closed arms. OCD, obsessive-compulsive disorder; QNP, quinpirole; OFT, open field test; EPM, elevated plus maze; DBS, deep brain stimulation; HPLC, high-performance liquid chromatography; MS, mass spectrometry; NAc, nucleus accumbens. The data are presented as the mean ± SEM; *n* = 8 vs. *n* = 92; ****P < 0.0001; ns: no significance.

The stimulation and recording micro-electrodes were constructed based on the semi-finished products (#366-080906-22, Alpha-Omega, Israel). The rats were placed on a stereotaxic apparatus after anesthetization with isoflurane (RWD Life Science, China). According to the stereotaxic atlas of Paxinos and Watson (6th edition), the micro-electrodes were implanted in the core of the left NAc (AP = + 2.00 mm, ML = + 1.60 mm, DV = −7.20 mm), as shown in Figure 3A. Moreover, 3 screws were placed on the skull as anchors for the ground wire of the micro-electrodes. The holes were drilled properly after the appropriate locations were measured and marked on the skull. A micro-electrode was attached to the holder and lowered into the NAc perpendicularly through the drilled hole slowly. The micro-electrode and screws were fixed securely with dental cement. After the operation, the rats were given penicillin (Sigma-Aldrich, US, 100 mg/kg, i.p.) and carprofen (Sigma-Aldrich, US, 10 mg/kg, i.p.) every day for 3 days.

### Stimulation and electrophysiological signals acquisition

After recovery, a stimulation program based on the most commonly used parameters was administrated to the rats in the NAc-DBS group. A bipolar biphasic current-controlled pulse that was rectangular in shape (90 μs negative/90 μs positive) and had a frequency of 130 Hz and current intensity of 150 μA was delivered for 1 h/day for 5 days. When the stimulation was finished, the *in vivo* neural electrophysiological signals were recorded by the AlphaLab SNR system (Alpha-Omega, Israel). The original signals were split into SPK and LFP activity by digitization at different sampling rates of 22000 and 1375 Hz, and band-pass filtering at 300–9000 and 0.5–200 Hz. The online-sorted SPK signals and the raw LFP signals were both saved for further offline analysis.

The SPK signals were analyzed with Spike2 (Cambridge Electronics Designs, UK) and NeuroExplorer (Nex Technologies, US), and the firing rate and ISI were analyzed to evaluate the firing pattern of the neurons in the NAc. The mean firing rate, which represented the extent of neuronal activity, was defined as the number of spikes divided by the duration of the period; the CV, which represented the whole dispersion, was defined as the standard deviation of the ISI divided by the mean ISI; and the AI, which provides information about the shape of the ISI distribution curves, was defined as the mode of the ISI divided by the mean ISI.

In addition, the LFP signals were analyzed with NeuroExplorer and its extended scripts in MATLAB 2017a (MathWorks, US), and time-frequency spectrogram analysis and PSD analysis were performed to assess the extracellular postsynaptic membrane potential changes in the NAc. It is worth noting that the division of different frequency bands in the present study was as follows: delta band (1–3 Hz), theta band (3–7 Hz), alpha band (7–12 Hz), beta band (12–30 Hz) and gamma band (30–80 Hz).

### Slice preparation and immunostaining

After the signals were collected, the rats in each group were sacrificed using a lethal dose of sodium pentobarbital. The rats were perfused with 150 mL 0.01 M phosphate-buffered saline (PBS) and 150 mL of 4% paraformaldehyde (PFA). Then, the rat brains were removed, and tissue blocks containing the NAc, VTA and RN were dissected out. Then, 4 μm coronal paraffin sections were prepared.

Ventral tegmental area (VTA) sections were used for TH immunofluorescence staining, and RN sections were used for TPH2 immunofluorescence staining. After heat-induced antigen retrieval in a water bath (97°C, 40 min) and citrate buffer (pH = 6.0), the sections were blocked with 5% bovine serum albumin (BSA) at 37°C for 1 h and incubated with primary antibodies (anti-TH, Abcam, UK, #ab6211, 1:1000; anti-TPH2, Abcam, UK, #ab288067, 1:300) at 4°C overnight. The sections were then washed with 0.01 M PBS (3 × 10 min) and incubated with the corresponding secondary antibodies (Alexa Fluor^®^ 568, Abcam, UK, #ab175692, 1:200) at 37°C for 1 h. The sections were incubated with DAPI (1:1000) at room temperature for 10 min and then washed with 0.01 M PBS (3 × 10 min).

Nucleus accumbens (NAc) sections were used for immunohistochemical staining of DRD1 and DRD2. After antigen retrieval as described above, endogenous peroxidase activity was blocked with 3% H_2_O_2_ at room temperature for 30 min. The sections were then blocked with 5% BSA (37°C, 1 h) and incubated with primary antibodies (anti-DRD1, Abcam, UK, #ab279713, 1:500; anti-DRD2, Affinity Biosciences, Australia, #DF10211, 1:100) at 4°C overnight. The sections were then washed with 0.01 M PBS (3 × 10 min) and incubated with the corresponding secondary antibodies (horseradish peroxidase-conjugated anti-rabbit secondary antibodies, Jackson, US, #111-035-003, 1:200) at 37°C for 1 h. Subsequently, the sections were incubated with DAB for chromogenic staining and then washed with 0.01 M PBS (3 × 10 min).

Immunofluorescence staining images were acquired with an inverted laser confocal microscope (Nikon, Japan, N-STORM & A1). Immunohistochemical staining images were acquired with a multispectral panoramic microscope (PerkinElmer, US, Vectra Polaris). Imaging parameters were kept consistent among sections for the same antibody. The immunofluorescence intensity was measured by NIS-Elements Analysis 5.21.00 (Nikon, Japan), and the integrated optical density (IOD) was measured by Image-Pro Plus (Media Cybernetics, US).

### Sample preparation for targeted metabolomics and data collection

After signals were collected, the rats in each group were decapitated, and then brain samples (the ipsilateral NAc) were rapidly harvested, placed in cryogenic vials and frozen in liquid nitrogen. The brain tissues (10 mg) were weighed and homogenized (4°C, 6.5 m/s, 4 × 30 s/cycle) in 200 μL methanol containing internal standards (−80°C, ^13^C5-Glutamic acid-^15^N/^13^C6-Glucose, Cambridge Isotope Laboratories, UK). Then, 800 μL methanol without internal standards (−80°C) was added, and the mixture was vortexed (4°C, 1500 rpm, 3 min). The samples were sonicated in an ultrasonic water bath (10 min), vortexed (4°C, 1500 rpm, 3 min) again and centrifuged (4°C, 13300 rpm, 15 min). The supernatants (800 μL) were transferred to new centrifuge tubes and dried in a centrifugal vacuum evaporator at 30°C. The samples were reconstituted in 500 μL of mobile phase A (water:acetonitrile = 9:1, 0.2% acetic acid, 10 mM ammonium acetate):mobile phase B (water:acetonitrile = 1:9, 0.2% acetic acid, 10 mM ammonium acetate) (3:7) containing ^13^C9-L-tyrosine-^15^N/^13^C1-lactate (Cambridge Isotope Laboratories, UK), vortexed for 10 min at 1500 rpm and centrifuged for 10 min at 13300 rpm (4°C).

An HPLC system (Shimadzu Corporation, Japan, LC-30AD) coupled with a triple quadrupole MS system (Sciex, US, Q-Trap 5500) equipped with a turbo ion spray source, including scheduled MRM positive mode and negative mode, was used for HPLC–MS analysis. Chromatographic separation was performed at 40°C on a Acquity UPLC BEH Amide column (1.7 μm, 2.1 × 100 mm, Waters, UK). The gradient elution was as follows: 0.0∼0.1 min: 10% A; 0.1∼1.5 min: 10% A; 1.5∼5.0 min: 55% A; 5.0∼10.0 min: 55% A; 10.0∼12.0 min: 10% B; and 12.0∼25.0 min: 10% A. The flow rate was 0.3 mL/min, the autosampler temperature was 6°C, and the sample injection volume was 2 μL for positive mode and 10 μL for negative mode. The ion source parameters: ion spray voltage: ±4000 V; temperature: 650°C; gas-1: 50 psi; gas-2: 40 psi; and curtain gas: 35 psi. The raw data were uploaded to MultiQuant software (Sciex, US, V2.0.3) for pretreatment. Then, data collected for the main neurotransmitters of interest were extracted for further processing.

### Statistical analysis

All the data are expressed as the means ± standard errors of the mean. Statistical analyses were conducted using SPSS 22. For comparison between two independent groups, the D’Agostino Pearson method was used to test normality, and homogeneity of variance was tested by F-test, then Student’s *t-*test was suitable to taken for analyzing. For comparisons among three independent groups, the D’Agostino Pearson method was used to test normality, and homogeneity of variance was tested by Brown-Forsythe method, then one-way analysis of variance (ANOVA) followed by Tukey’s *post hoc* test was suitable to taken for analyzing. *P* < 0.05 was considered significant, and *P* < 0.01 was considered highly significant.

## Results

### Compulsive checking behavior and anxiety behavior

After 10 injections, rats administered QNP exhibited stable compulsive check behavior. Moreover, unlike the saline-treated rats, the QNP-treated rats did not explore the whole open field arena and focused on specific objects as their home base (Figure 3B). Compared with the control rats, the QNP-treated rats exhibited increases in the number of home base visits and the total distance traveled (*n* = 8 vs. *n* = 92) (Figure 3C). However, there were no significant differences in anxiety-related behaviors (Figures 3D, E).

### Alterations in SPK activity in the NAc

**FIGURE 4:**
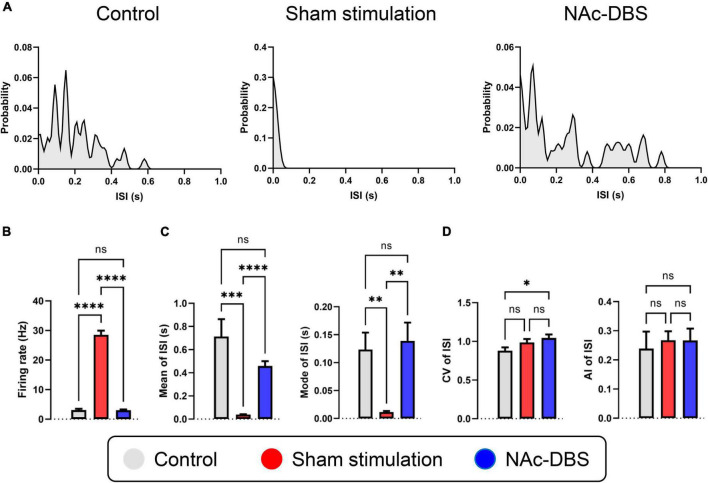
Alterations in the firing rate and firing patterns of neurons in the NAc. **(A)** Representative distribution curves of interspike intervals of control, sham stimulation and NAc-DBS groups. **(B)** The bar graph represents the mean level of firing rates. **(C)** The bar graphs represent the mean and the mode of ISI. **(D)** The bar graphs represent the level of CV and AI of ISI. ISI, interspike interval; CV, coefficient of variance; AI, asymmetry index. The data are presented as the mean ± SEM; *n* = 24 vs. *n* = 47 vs. *n* = 48; ****P < 0.0001, ***P < 0.001, **P < 0.01, *P < 0.05; ns, no significance.

A total of 24 neurons from the control group, 47 neurons from the sham stimulation group and 48 neurons from the NAc-DBS group were recorded. The neurons from each group were classified as a single type based on the SPK duration and firing properties. Representative distribution curves of ISI are shown in Figure 4A. The firing rate was obviously increased in rats in the sham stimulation group compared with those in the control group, and the firing rate in the NAc decreased after application of NAc-DBS (Figure 4B). The ISI mean and mode were lower in the sham stimulation group than in the control group and were restored to a certain extent following NAc-DBS (Figure 4C). Additionally, we found that the CV of neurons in the NAc-DBS group was increased compared with that of neurons in the control group but did not differ from that of neurons in the sham stimulation group. Moreover, no significant difference in the AI among three groups was found at present (Figure 4D).

### Alterations in LFP activity in the NAc

**FIGURE 5:**
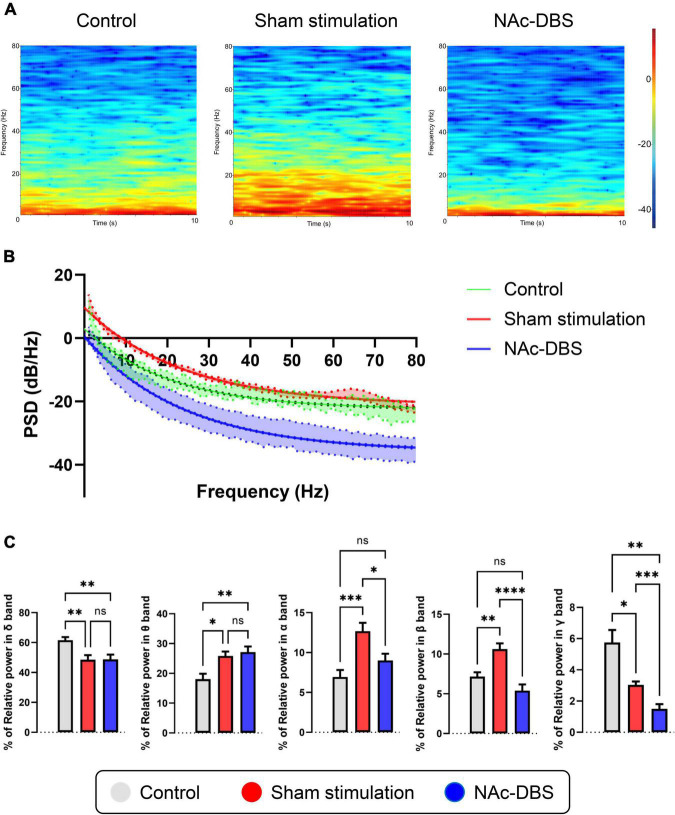
Illustrations of the difference in LFP activity in the NAc. **(A)** Representative time-frequency spectrograms in 10 s showing the power level of LFP from 1 to 80 Hz in the NAc of the control, sham stimulation and NAc-DBS groups. **(B)** The mean PSD curve with 95% confidence interval from 1 to 80 Hz. **(C)** The bar graphs represent the mean level of relative LFP powers of the delta band, theta band, alpha band, beta band and gamma band in the NAc in the different group. PSD, power spectral density. The data are presented as the mean ± SEM; *n* = 8 vs. *n* = 34 vs. *n* = 28; ****P < 0.0001, ***P < 0.001, **P < 0.01, *P < 0.05; ns, no significance.

Local field potentials (LFP) signals were obtained from each 10 s segment of recordings from the NAc in the control group (*n* = 8), the sham stimulation group (*n* = 34) and the NAc-DBS group (*n* = 28). Visual inspection of representative time-frequency spectrograms and averaged PSD curves with 95% confidence intervals showed that the power of low frequency bands was increased in the OCD model group compared with the control group and that the overall power levels were decreased in rats treated with NAc-DBS (Figures 5A, B). For further evaluation of the PSD in the NAc, the relative powers of the different bands were calculated and compared (Figure 5C). Compared with those in the control group, rats in the sham stimulation group showed higher relative power in the theta band, alpha band and beta band oscillatory activity in the NAc, and these changes in the alpha band and beta band were reduced in rats treated with NAc-DBS. However, there was no difference in the theta band after NAc-DBS. The rats in the sham stimulation group and NAc-DBS group showed a lower relative power in the delta band than the control group, but there was no noticeable difference between the sham stimulation and NAc-DBS groups. The relative power of the gamma band (30-80 Hz) was lower in the sham stimulation group than in the control group and it was even lower in the NAc-DBS group.

### Changes in dopaminergic neurons in the VTA and serotonergic neurons in the RN

**FIGURE 6:**
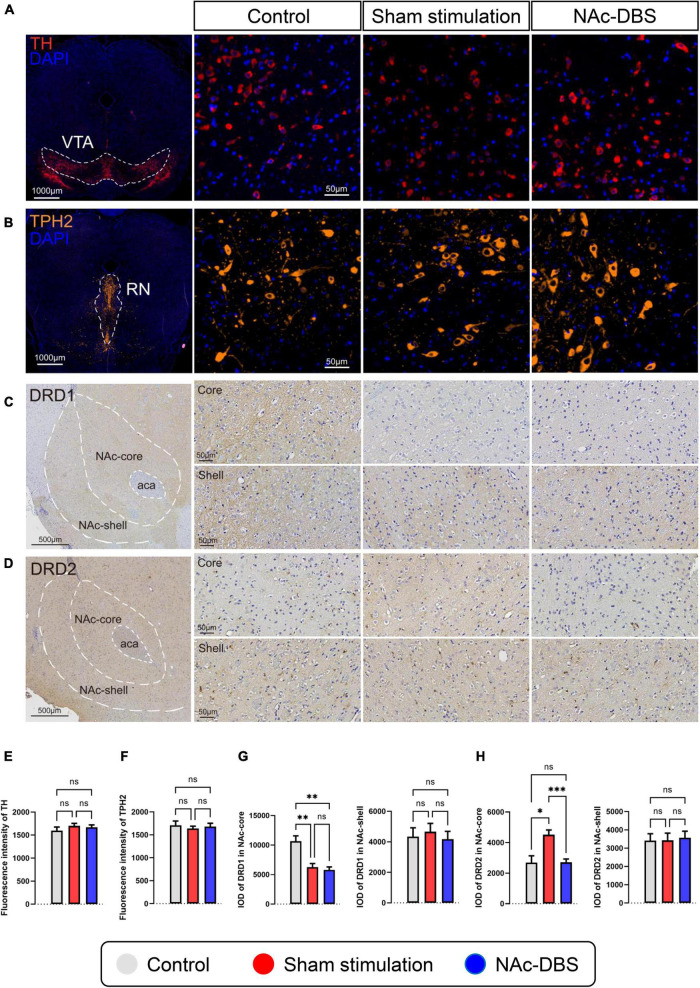
Differences in pathological sections among the control, sham stimulation and NAc-DBS groups. **(A)** Immunofluorescence staining for TH was performed to label all dopaminergic neurons in the VTA. **(B)** Immunofluorescence staining for TPH2 was performed to label all serotonergic neurons in the RN. **(C,D)** Immunohistochemical staining for DRD1 and DRD2 in the NAc. The top image is the NAc core, and the bottom image is the NAc shell. **(E)** The bar graph represents the mean level of the immunofluorescence intensity of TH in the VTA. **(F)** The bar graph represents the mean level of the immunofluorescence intensity of TPH2 in the RN. **(G)** The bar graphs represent the mean level of IOD of DRD1 in the core and shell of the NAc. **(H)** The bar graphs represent the mean level of IOD of DRD2 in the core and shell of the NAc. VTA, ventral tegmental area; TH, tyrosine hydroxylase; RN, raphe nuclei; TPH2, tryptophan hydroxylase-2; NAc, nucleus accumbens; DRD1, dopamine receptor-1; DRD2, dopamine receptor-2; IOD, integrated optical density. The data are presented as the mean ± SEM; *n* = 10 vs. *n* = 19 vs. *n* = 19 (immunofluorescence); *n* = 8 vs. *n* = 17 vs. *n* = 15 (immunohistochemistry); ***P < 0.001, **P < 0.01, *P < 0.05; ns, no significance.

Staining for TH was conducted to visualize dopaminergic neurons in the VTA, and staining for TPH2 was performed to visualize serotonergic neurons in the RN. As shown in the immunofluorescence images in Figures 6A, B, no noticeable difference in cell morphology was observed among the different groups. Furthermore, analysis of fluorescence intensity did not reveal any significant difference (*n* = 10 vs. *n* = 19 vs. *n* = 19) (Figures 6E, F).

### Changes in DRD1 and DRD2 expression in the NAc

The core part and shell part of the NAc, which exhibit diffuse expression of DRD1 and DRD2, respectively, were observed separately by immunohistochemical staining. No obvious differences in cell morphology or density were found among the different groups. The intensity of DRD1 staining in the NAc core was weaker in the sham stimulation group and NAc-DBS group than in the control group, and the intensity of DRD2 staining in the NAc core was stronger in the sham stimulation group than in the other two groups (Figures 6C, D upper). However, there was no difference in staining intensity in the NAc shell (Figures 6C, D lower). Moreover, analysis of the IOD revealed a consistent trend (*n* = 8 vs. *n* = 17 vs. *n* = 15) (Figures 6G, H).

### Relative changes of in neurotransmitter levels in the NAc

A total of 11 samples from the control group, 23 samples from the sham stimulation group and 21 samples from the NAc-DBS group were collected. The level of DA in the NAc was increased in the sham stimulation group compared to the control group and decreased in rats treated with NAc-DBS (Figure 1B). The level of 5-HT in the NAc decreased in the NAc-DBS stimulation group compared to the sham stimulation group but did not differ between the control group and the sham stimulation group (Figure 1D). Moreover, we did not detect any significant differences in the levels of histamine and ACh in the NAc among the different groups (Figures 1F, H). Glu and GABA levels were increased in the sham stimulation group compared to the control group and restored to normal levels after NAc-DBS (Figures 1J, L).

## Discussion

The hypothesis of this study is that there were some underlying disturbance both in the neural electrophysiological activities and neurotransmitters activities in the NAc, and NAc-DBS could modulate and correct these disturbance both in electrical and chemical ways. To determine the potential mechanism by which NAc-DBS can alleviate QNP-induced OCD in rats, we systematically investigated the effect of NAc-DBS on electrophysiological activities *in vivo* and relevant neurotransmitter activities. This study could be the first one to systematically explore the electrophysiological characteristics of the NAc in OCD and DBS conditions not only from LFP but also SPK, and it could also be the first one to roundly explore the neurotransmitter changes in the NAc in OCD and DBS conditions. In general, successful induction of OCD in rats upon chronic administration of QNP was confirmed by relevant behavioral tests. A micro-electrode was implanted into the core part of NAc to obtain information about SPK and LFP in the NAc under different conditions, and the levels of neurotransmitters of interest at the tissue level were measured through HPLC–MS.

This study provides direct evidence that OCD and high-frequency DBS of the NAc core have different effects on neuronal firing patterns and synchronous fluctuations in large populations of neurons in the NAc. The SPK activity of NAc neurons was significantly different between rats in the sham stimulation group and rats in the control group, and the changes induced by QNP administration were substantially reversed by the application of NAc-DBS. The SPK firing rate was markedly increased and the ISI was obviously decreased in OCD model rats, indicating that the NAc was hyperactive, which is a novel finding. Moreover, the SPK firing rate and ISI were altered toward normal levels when electrical stimulation was applied; this finding is in accordance with a previous study that used brain slices (Xie et al., 2014). In the present study, the firing pattern of NAc neurons, as represented by the mean and mode ISI, CV and AI of ISI, was also significantly affected by QNP administration and NAc-DBS.

Abnormalities in the NAc were also revealed by analysis of LFP activity. Our data showed that the low-frequency PSD (especially from 1 to 30 Hz) was enhanced in rats in the sham stimulation group compared to those in the control group but that the high-frequency PSD was not obviously changed; this alteration could be used as a pathological biomarker for OCD. Interestingly, upon administration of NAc-DBS, of the entire PSD, both the low-frequency PSD and high-frequency PSD, was decreased markedly. Through relative power analysis of different frequency bands, more meaningful details were found. Compared with those in the control group, the rats in the sham stimulation group presented higher relative power in the theta band, alpha band and beta band but lower relative power in the delta band and gamma band. These findings provide valuable information for identifying OCD-related biomarkers. After NAc-DBS, the relative power of the alpha band, beta band and gamma band decreased, but there was no significant difference in delta band and theta band. These findings reveal that high-frequency electrical stimulation of the NAc may result in special patterns of neuronal activity, which could be a potential basis for closed-loop stimulation in the future. The findings of this study support previous clinical research. In a study in which DBS of the ventral capsule (VC)/ventral striatum (VS) was applied, the effect of DBS was correlated with the activity of the alpha, beta and gamma bands (Olsen et al., 2020). Another study in which DBS of the bed nucleus of the stria terminalis (BNST) was administered found that the normalized delta, beta and gamma band power in the right BNST was specifically correlated with compulsive behaviors (Wu et al., 2016). There is also some evidence suggesting that the activities of the alpha band and beta band are related to depression (Neumann et al., 2014). In addition, some research has indicated that oscillatory activity of the theta band in the striatal area may be a potential biomarker for OCD (Neumann et al., 2014; Miller et al., 2019; Schwabe et al., 2021), and studies in which DBS of the subthalamic nucleus (STN) was applied to treat OCD have shown that theta band activity is correlated with the severity of OCD symptoms (Rappel et al., 2018; Buot et al., 2021). Our data are generally consistent with these previous results and provide more detailed and comprehensive information about SPK and LFP activity in the NAc in OCD and after NAc-DBS, filling a knowledge gap.

The present study also showed that OCD and NAc-DBS have different influences on the neurotransmitter system in the NAc. Immunofluorescence revealed that dopaminergic neurons in the VTA, the main dopaminergic projections to the NAc, did not exhibit changes. However, we found that the level of DA in the NAc was increased in the sham stimulation group and decreased upon NAc-DBS. In addition, we found that the expression of DRD1 was decreased and the expression of DRD2 was increased in the NAc core in the sham stimulation group; this change did not occur in the NAc shell. This finding is in line with the previous research (Varatharajan et al., 2015; Servaes et al., 2019), however, in a study in which the NAc shell was stimulated rather than the NAc core, the level of DA was increased after DBS (Sesia et al., 2010). This may have been due to the differences in the functions of the core and shell. Immunofluorescence revealed no change in serotonergic neurons in the RN, but the level of 5-HT in the NAc was reduced upon NAc-DBS treatment. This finding is inconsistent with the results of previous research (Sesia et al., 2010), possibly due to differences in stimulation location. Furthermore, an early study in which unilateral DBS of the NAc core was applied showed no change in local monoamine release (van Dijk et al., 2011), there may be many reasons for this inconsistency, especially differences in stimulation parameters. Interestingly, in another relevant study, DA, 5-HT and noradrenaline levels in the prefrontal cortex (PFC) were increased upon NAc-DBS (van Dijk et al., 2012). This finding indicates that stimulation of a particular brain area may have different effects on the level of the same neurotransmitter in different brain regions. No significant alterations in the level of histamine or ACh was observed in this study; nevertheless, these two substances have important functions in the NAc. The NAc core receives direct histaminergic projections from the TMN, and the symptoms of OCD and anxiety are influenced by the activity of the presynaptic H3 histamine receptor on glutamatergic afferent terminals from the prelimbic PFC to the core of the NAc (Zhang et al., 2020). More detailed information about this pathway is needed. Moreover, cholinergic interneurons are essential components of the NAc (Xie et al., 2014), they generally exert mutual antagonistic effects with DA, but whether this is the case in the NAc is still unclear. Finally, the levels of Glu and GABA should be evaluated because an imbalance between Glu and GABA levels is thought to be responsible for neuronal hyperexcitability in neuropsychiatric disorders. In the present study, we found that both Glu levels and GABA levels in the NAc were increased after sham stimulation and that this change could be markedly reversed through NAc-DBS. However, the results of this study are somewhat different from those of previous reports. Glu is the most prominent excitatory amino acid in the brain, and elevation of the extracellular level of Glu is thought to be related to many neuropsychiatric disorders (Dahchour et al., 2000; Tokuyama et al., 2001; Hanna et al., 2002; Arnold et al., 2004). Consistent with this research, some studies have shown that the level of Glu is increased in OCD (Egashira et al., 2008; Yan et al., 2013). Furthermore, GABA is the most important inhibitory amino acid in the brain, and it is commonly accepted that GABAergic medium spiny neurons projecting to the VP are inhibited by excess DA release in the NAc, which is a key node in reward circuitry (Smith et al., 2009). Other research has demonstrated that the extracellular level of GABA in the NAc decreases when reward circuitry is disturbed and increases upon DBS (Yan et al., 2013; Varatharajan et al., 2015), which is not in line with our results. Notably, GABAergic projection neurons are the main neurons in the NAc (Smith and Villalba, 2008), but extracellular GABA in the NAc is predominantly derived from VS and VTA collaterals that project to the VP (Brog et al., 1993; Pennartz et al., 1994). Differences in methods between these previous studies (*in situ* microdialysis) and the present study (tissue homogenates) may have caused the inconsistencies in findings, or there may be deeper mechanisms involved that need to be further explored.

There are some limitations in this study. First, the micro-electrodes were just implanted into the NAc, the neural electrophysiological information we could obtain was confined to single region. The follow-up studies which have more comprehensive recording of multiple brain regions may reveal more valuable hints. On the other hand, the analyzing of neurotransmitters was based on tissue homogenates which has less temporal resolution and stable performance, like the first one, the analyzing of neurotransmitters should be also expanded to more areas simultaneously to form a more overall view to brain network in OCD and DBS conditions. Last but not least, the applying of evidences obtained from rats to humans truly should be carefully evaluated. Eventhough there could be some possible similar mechanisms, the functioning of the human mind compared to that of rat is still different. More in-depth research at primate especially human level is necessary.

In conclusion, our study shows that DBS of the NAc core apparently alters neuronal firing patterns as well as LFP activity in the NAc. Moreover, NAc-DBS decreases monoamine and amino acid neurotransmitter levels in the NAc, reversing the effect of OCD. All these results suggest that high-frequency electrical stimulation of the NAc core inhibits the activity of neurons in the NAc, and this change could underlie the ability of NAc-DBS to alleviate OCD. All these findings of this study provide a chance to exert the full potential of the application of NAc-DBS. Moreover, these findings also provide a new insight into the possible candidate biomarkers for OCD and electrochemical targets for developing closed-loop neurostimulation.

## Data availability statement

The original contributions presented in this study are included in the article/supplementary material, further inquiries can be directed to the corresponding author.

## Ethics statement

All the animal experimental and care procedures were approved by the Biomedical Research Ethics Committee of West China Hospital (Protocol Number: 20211434A).

## Author contributions

YS, JZ, and WW designed the research. YS, MW, LX, LG, LB, and BS conducted the research. YS, LG, and WZ performed statistical analysis. YS, MW, and LX wrote the manuscript. BL, YX, WP, and WW reviewed and edited the manuscript. WW supervised the work, acquired funding, and had primary responsibility for the final content. All authors contributed to the manuscript writing.
